# Novel features of ARS selection in budding yeast *Lachancea kluyveri*

**DOI:** 10.1186/1471-2164-12-633

**Published:** 2011-12-28

**Authors:** Ivan Liachko, Emi Tanaka, Katherine Cox, Shau Chee Claire Chung, Lu Yang, Arael Seher, Lindsay Hallas, Eugene Cha, Gina Kang, Heather Pace, Jasmine Barrow, Maki Inada, Bik-Kwoon Tye, Uri Keich

**Affiliations:** 1 Department of Genome Sciences, University of Washington, Seattle, WA, USA; 2 School of Mathematics and Statistics, University of Sydney, Sydney, Australia; 3 Department of Biology, Ithaca College, Ithaca, NY, USA; 4 Department of Molecular Biology and Genetics, Cornell University, Ithaca, NY, USA

## Abstract

**Background:**

The characterization of DNA replication origins in yeast has shed much light on the mechanisms of initiation of DNA replication. However, very little is known about the evolution of origins or the evolution of mechanisms through which origins are recognized by the initiation machinery. This lack of understanding is largely due to the vast evolutionary distances between model organisms in which origins have been examined.

**Results:**

In this study we have isolated and characterized autonomously replicating sequences (ARSs) in *Lachancea kluyveri *- a pre-whole genome duplication (WGD) budding yeast. Through a combination of experimental work and rigorous computational analysis, we show that *L. kluyveri *ARSs require a sequence that is similar but much longer than the ARS Consensus Sequence well defined in *Saccharomyces cerevisiae*. Moreover, compared with *S. cerevisiae *and *K. lactis*, the replication licensing machinery in *L. kluyveri *seems more tolerant to variations in the ARS sequence composition. It is able to initiate replication from almost all *S. cerevisiae *ARSs tested and most *Kluyveromyces lactis *ARSs. In contrast, only about half of the *L. kluyveri *ARSs function in *S. cerevisiae *and less than 10% function in *K. lactis*.

**Conclusions:**

Our findings demonstrate a replication initiation system with novel features and underscore the functional diversity within the budding yeasts. Furthermore, we have developed new approaches for analyzing biologically functional DNA sequences with ill-defined motifs.

## Background

Eukaryotic DNA replication initiates at loci known as origins of replication. In budding and fission yeast origins are short sequences (< 1 kb) that allow autonomous replication of episomal plasmids [1-7]. This property allows for precise identification and manipulation of replication origins, also known as autonomously replicating sequences (ARSs). Studies in *Saccharomyces cerevisiae *have identified a conserved ARS consensus sequence (ACS) [3,8-10]. This 11 bp motif is necessary, but not sufficient for the initiation of DNA replication. Other factors, such as flanking "B elements" [11-13], nucleosome exclusion sites [14,15], local transcription activity [16,17], and elements influencing the helical stability of DNA [18,19] have also been shown to play a role in origin function. We recently showed that by using a 33 bp long (extended) ACS motif we are able to predict *S. cerevisiae *ARSs with a fairly high accuracy [20]. This high accuracy was achieved using a PWM (position weight matrix) representation of the motif rather than the consensus representation that is commonly used for the "canonical" 11 bp ACS motif. Presumably the extended motif bundles the core ACS with some of its flanking elements [21]. In contrast, in fission yeast *Schizosaccharomyces pombe *and in metazoans, no consensus motifs have been identified to date and replication origins are selected in a mostly stochastic manner at regions of AT rich DNA sequences [22-26].

In *S. cerevisiae *origins the ACS is bound by the Origin Recognition Complex (ORC) which coordinates the assembly of the pre-replication complex (pre-RC) and the subsequent initiation of DNA synthesis [3,13]. However, while ORC binding is required for replication licensing, ORC also binds to regions where initiation does not take place. The strict 11 bp ACS motif is present thousands of times throughout the genome and only a subset of these 11 bp ACS motifs are bound by ORC and an even smaller subset correspond to active origins [27,28]. In addition, not all origins fire in every cell division and a temporal program along with stochastic effects controls the timing of origin firing in S phase [29,30]. In *S. pombe*, Orp4, one of the subunits of ORC contains a unique AT-hook domain that allows ORC to bind stochastically to regions of AT-rich DNA [23,31,32]. This stochastic nature of ORC binding contributes to an increased number of potential origin sites, while simultaneously lowering the probability of any given site firing within a specific cell cycle [24,25]. *S. pombe *origin sites can be most accurately predicted using an approach that seeks A-T rich islands [33]. In metazoan cells, origin sites are much larger and less well defined than in yeast. Replication initiation is thought to be largely stochastic, and in some systems random DNA sequence serves as an initiation site [33-37].

Due to the large evolutionary distance between *S. cerevisiae*, *S. pombe *[38], and metazoans, little is understood about the evolution of origins and the mechanisms of origin selection. Since *S. cerevisiae *origins share a conserved motif, while other model organisms do not, it is tempting to speculate that motif-driven replication origins are unique to the *Saccharomyces *yeasts and that species more similar to *S. pombe *would have more stochastic initiation mechanisms. However, a recent study in a pre-whole genome duplication (WGD, [39]) yeast *Kluyveromyces lactis *has revealed origin structure that relies on a 50 bp ACS motif that does not structurally resemble the *S. cerevisiae *ACS, nor the AT rich domains of *S. pombe *origins [20,40-42]. This motif is necessary and generally sufficient for *K. lactis *ARS function and had been used to dependably predict genome-wide origin locations with a much higher accuracy than is possible in *S. cerevisiae*. Notably, we have observed that *K. lactis *ARSs rarely function in *S. cerevisiae *and vice versa [20]. This finding suggests that though both species have retained a motif-driven origin selection process, they have significantly diverged in the motifs used. Since *K. lactis *is much more closely related to *S. cerevisiae *and the other budding yeasts than to S. pombe, this finding suggests a diversity of origin structure as well as origin selection mechanisms among the budding yeasts.

To further explore the mechanisms of DNA replication initiation among the budding yeast species, we have investigated the structure of ARSs from another pre-WGD yeast - *Lachancea (Saccharomyces) kluyveri *whose genome has been recently annotated [43,44]. This species is thought to have diverged from the *Saccharomyces *lineage ~150 million years ago and has an 11.3 Mb genome organized into 8 chromosomes. In this study we have used a random genomic screen to isolate large numbers of ARSs from *L. kluyveri*. We found that *L. kluyveri *is characterized by a permissive mechanism of ARS selection. All *S. cerevisiae *ARSs and most *K. lactis *ARSs function in *L. kluyveri*. However, fewer than half of *L. kluyveri *ARSs tested function in *S. cerevisiae *and very few function in *K. lactis*, suggesting that these organisms have more stringent requirements for a functional ARS than *L. kluyveri*. We also identified a putative 9 bp long *L. kluyveri *ACS motif that is similar to the *S. cerevisiae *ACS but shorter. However, whereas ARS activity can be predicted almost perfectly using a 50 bp ACS motif in *K. lactis *and with fairly high accuracy using a 33 bp ACS motif in *S. cerevisiae*, ARS activity in *L. kluyveri *seems to be determined by a much longer sequence that includes the 9 bp ACS. Even with this extended sequence, prediction of ARS function remains significantly less successful when compared to the other two yeast species. Taken together, our findings suggest that *L. kluyveri *has an initiation mechanism bearing features of both motif-driven and stochastic models.

## Results

### The Isolation of *L. kluyveri *ARSs

**Figure 1:**
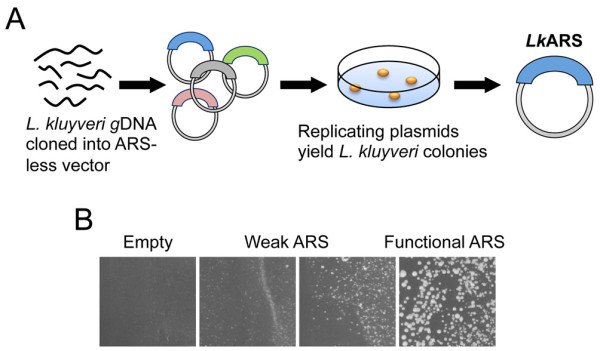
**Screen to isolate *L. kluyveri *ARSs**. (A) *L. kluyveri *genomic DNA was fragmented with MboI and ligated into the pIL07 vector. The resultant libraries were transformed into *L. kluyveri *strain FM628 and ARS plasmids were isolated from resulting colonies. (B) Representative colony sizes of plasmids showing ARS activity or the lack thereof.

In order to identify functional ARSs in *L. kluyveri*, we performed a random ARS screen as described previously [7,20] (Figure 1A). Genomic DNA from *L. kluyveri *was digested to completion with MboI (a 4-cutter restriction enzyme) and ligated into an ARS-less vector bearing a *URA3 *marker. Ligation mixtures were transformed into *E. coli *and the resultant colonies were pooled to construct genomic plasmid libraries. These genomic libraries were transformed into *L. kluyveri *and plated on selective media lacking uracil. Cells bearing ARS plasmids are able to propagate the plasmids and form colonies, while non-replicating plasmids do not yield colonies (Figure 1B). Plasmids from robust yeast colonies were isolated, sequenced, and transformed back into *L. kluyveri *to confirm ARS function. Using this approach we isolated 221 plasmids, from which we selected 84 as unique, non-overlapping sequences that unambiguously mapped to a single locus in *L. kluyveri*. From the set of 84 unique *Lk*ARSs, 69 localize to a single intergene, 10 overlap two consecutive intergenes (the insert spanning an entire gene), and 5 lie entirely within the ORF of an annotated gene (each fragment lies within a different gene). Two *Lk*ARSs are subtelomeric and 14 overlap with at least a single tRNA gene.

### *L. kluyveri *has permissive origin selection determinants

**Figure 2:**
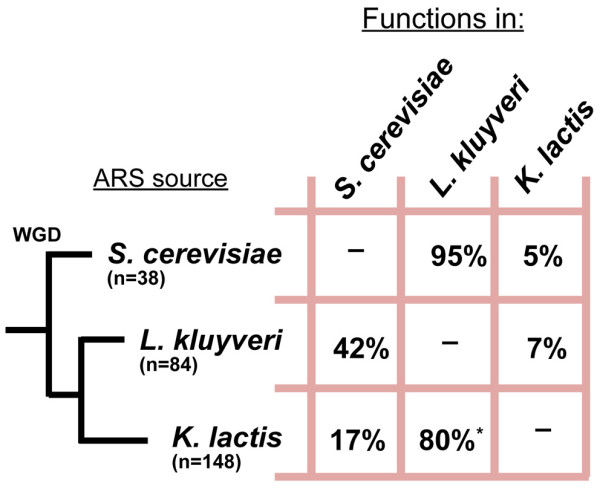
***L. kluyveri *has a permissive mechanism of ARS selection relative to *S. cerevisiae *and *K. lactis*. *Sc*ARS **[20], ***Lk*ARS, and *Kl*ARS **[20]**plasmids were transformed into *S. cerevisiae*, *L. kluyveri*, and *K. lactis *and assayed for ARS function in the different species**. The 'ARS source' column denotes the origin of the ARS, while the 'functions in' column denotes proportion of ARSs that are functional in the listed species. *: of the 80%, 20% of show weak ARS activity while 60% show strong ARS activity in *L. kluyveri*. 'WGD' denotes the whole genome duplication event leading to the *S. cerevisiae *lineage.

Previous work in *K. lactis *showed a striking divergence of the *Kl*ACS motif from the canonical *Sc*ACS [20,40,42,45]. This difference coincides with the inability of the *S. cerevisiae *DNA replication machinery to initiate replication from the majority of *Kl*ARSs and vice versa. To gain insight into the similarities between the ARS recognition mechanism of *L. kluyveri *and those of *S. cerevisiae *and *K. lactis*, we tested the *Lk*ARSs for function in the two other species as well as tested *Sc*ARSs and *Kl*ARSs for function in *L. kluyveri *(Figure 2, Additional File 1, Tables S1 & S2). The results of these experiments revealed a striking diversity in ARS recognition mechanisms among these three species.

*K. lactis *seems to have the most selective ARS recognition mechanism accepting only 5-7% of the ARSs from the other 2 species. *L. kluyveri *appears to have the most permissive mechanism: 80% of the 148 *Kl*ARSs function in *L. kluyveri *(28% of which display weak ARS function) compared with only 17% *Kl*ARSs that function in *S. cerevisiae*, in addition, 95% of *Sc*ARSs function in *L. kluyveri *compared with only 42% of *Lk*ARSs that function in *S. cerevisiae*. *L. kluyveri *and *S. cerevisiae *ARSs apparently share greater similarity to one another than to *K. lactis *ARSs: 42% of *Lk*ARSs tested function in *S. cerevisiae *versus only 17% of the *Kl*ARSs and 95% of *Sc*ARSs function in *L. kluyveri *versus 80% of the *Kl*ARSs (of which 28% exhibit only weak functionality). Finally, a common sequence element appears to be critical for enabling *Kl*ARSs to function in both *L. kluyveri *and *S. cerevisiae *because all 25 *Kl*ARSs that function in *S. cerevisiae *also function in *L. kluyveri *(p-value of 0.002). In contrast, the sequence elements of *Sc*ARSs or *Lk*ARSs critical for activity in the other two species appear to be independent as the proportion of *Sc*ARSs that function in *L. kluyveri *and *K. lactis *and the proportion of *Lk*ARSs that function in both *S. cerevisiae *and *K. lactis*, appear to occur by chance (p-values of 0.48 and 0.90) (see the Methods section for details). This result again suggests a closer relatedness between the origin recognition machinery of *S. cerevisiae *and *L. kluyveri *than the other pairwise relationship. This relationship is unexpected given that *K. lactis *and *L. kluyveri *are more closely related to each other than to *S. cerevisiae *[46,47].

### Identification of the *Lk*ARS Consensus Sequence

**Figure 3:**
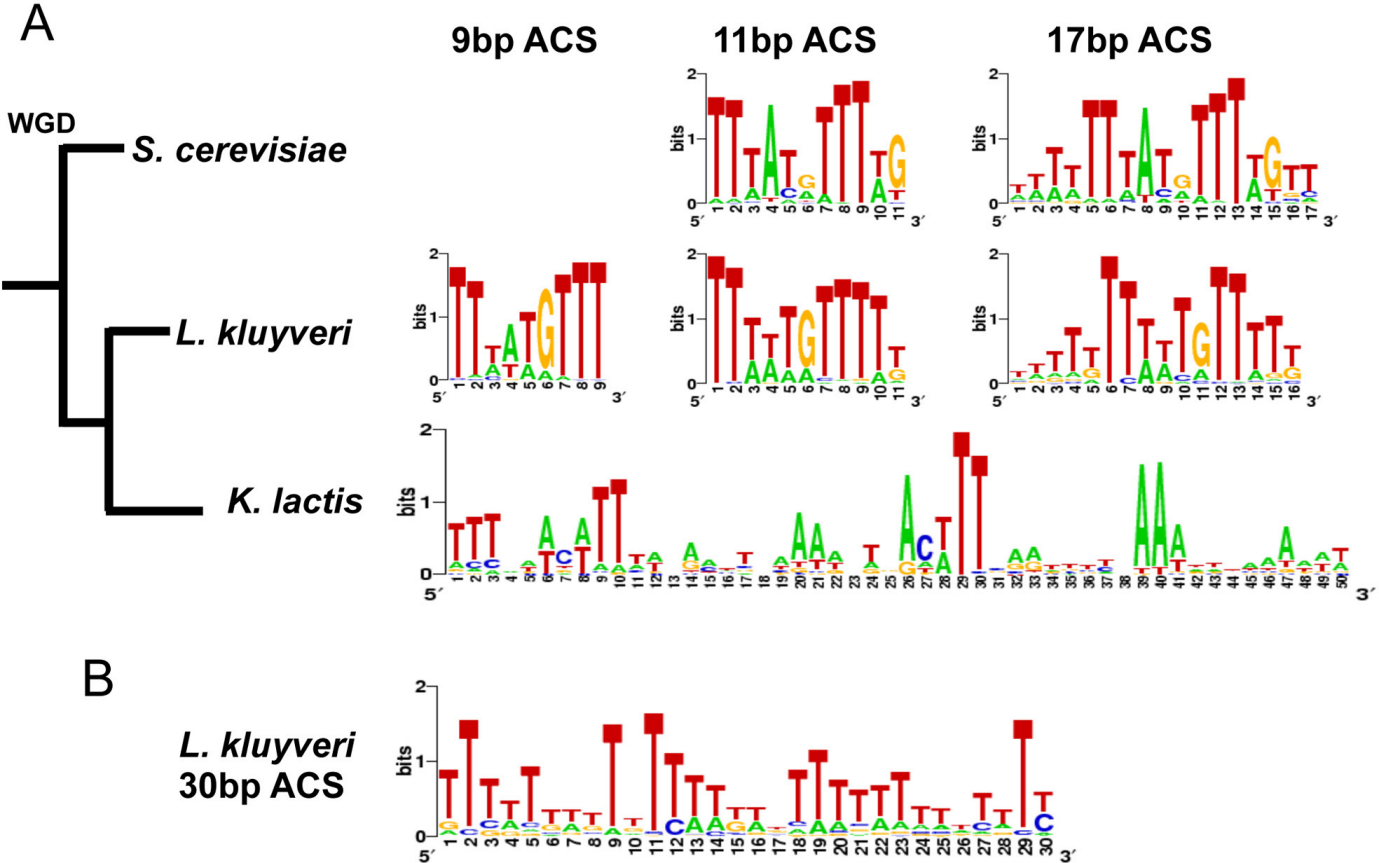
**Identification of the *Lk*ACS motif**. (A) Position Weighted Matrix logos of putative ACS motifs for *S. cerevisiae*, *L. kluyveri *and *K. lactis*. 'WGD' denotes the whole genome duplication event leading to the *S. cerevisiae *lineage. (B) Sequence logo of the statistically significant 30 bp motif found by GIMSAN in the set of 84 native *L. kluyveri *ARSs.

Our findings raise questions about the molecular determinants of ARS function in these three species. Are the observed differences in the level of ARS selectivity among the three yeast species reflected in the *cis *elements that are associated with replication initiation? Similarly, can the higher degree of ARS interchangeability between *L. kluyveri *and *S. cerevisiae *than between either one of these two and *K. lactis *be explained by specific DNA sequence elements? In order to address these possibilities we first need to identify the sequence determinants of ARS activity. We have previously shown that the 50 bp *K. lactis *ACS motif, as well as the 33 bp *S. cerevisiae *ACS motif, are particularly effective predictors of ARS functionality in their respective species [20]. To identify conserved DNA elements required for *Lk*ARS function we applied the motif finder GIMSAN [48], noted for its reliable significance analysis, to the set of 84 unique native *Lk*ARSs. GIMSAN was instructed to look for motifs of various widths (7 bp-17 bp, 25 bp, 30 bp, 40 bp, 50 bp). For most of these widths it found a motif that it deemed to be statistically highly significant. However, unlike in *S. cerevisiae *and *K. lactis *where the longer motifs clearly include the shorter motifs, the relationship between the significant *L. kluyveri *motifs of different lengths is not always clear. Specifically, while the motifs of lengths 8 bp-17 bp exhibit the same compatibility observed in the other two species across the entire range of lengths (e.g. the *Lk*ACS motifs of lengths 9 and 11 in Figure 3A), the relation of these shorter motifs to the highly significant longer motifs, which are largely T-rich, is no longer clear (e.g. compare the motifs of lengths 9 and 11 in Figure 3A with the motif of length 30 in Figure 3B).

Since the longer motifs are largely T-rich, we tested the sufficiency of A/T rich DNA for *Lk*ARS function. We cloned into our ARS-less vector a sequence of 25 and of 50 consecutive T nucleotides. When tested for ARS function, none of these plasmids had ARS activity in *L. kluyveri *(nor in the other two species). This result suggests that despite general T-richness of the *Lk*ACS, the functionally relevant motif contains information not captured by a stretch of A or T nucleotides alone.

We note that all the related *L. kluyveri *motifs in the range of 8 bp-17 bp show some distinct resemblance to the *Sc*ACS motif. At the same time the longer, 25-50 bp, motifs do not exhibit obvious resemblance to either the *Kl*ACS or the *Sc*ACS (Figure 3). While it is not necessary that the *Lk*ACS should resemble either the *Sc*ACS or the *Kl*ACS, if such resemblance is found it may lend some credence to the putative *L. kluyveri *motif. This resemblance to the *Sc*ACS motif is particularly important here as it may help explain our finding that *L. kluyveri *and *S. cerevisiae *have a much higher rate of mutual acceptance of ARSs than any one of them has with *K. lactis*, as well as explain the significant number of functional *Kl*ARSs they share. While this argument suggests that the 8-17 bp motifs are more relevant for ARS function than the longer ones, more data is needed to determine the most relevant one among them.

### Truncation and Mutagenesis Analysis

**Figure 4:**
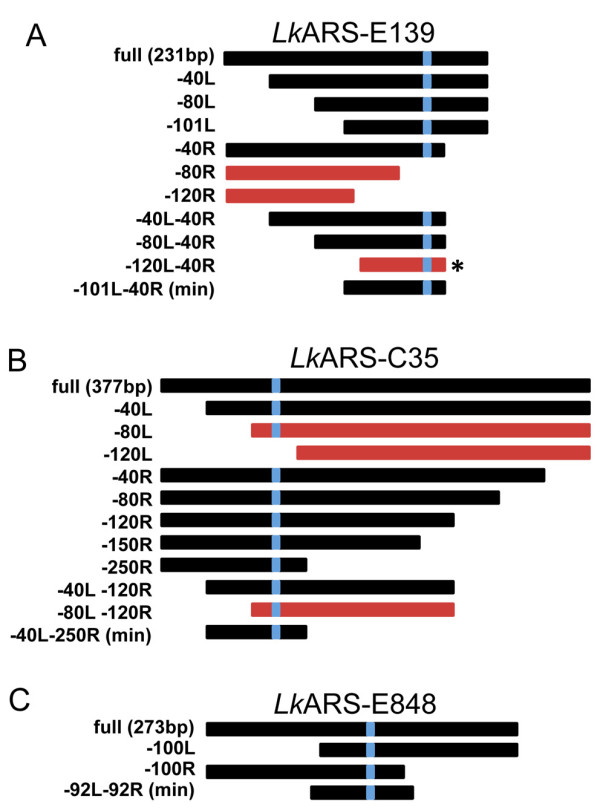
**Truncation of *Lk*ARSs to narrow down essential functional regions**. (A-C) Sub-fragments of three *Lk*ARSs (two more shown in Additional File 2, Figure S1) were cloned and tested for ARS function. Black boxes represent functional fragments. Red boxes represent non-functional fragments. For each ARS, the position of the best match to the 9 bp *Lk*ACS is indicated by a blue box. The extent of the truncation in basepairs is indicated on the left of the graphics (L = truncated from the left, R = truncated from the right). The length of the original full-length fragment isolated from the screen is indicated next to the first fragment from the top. *: This fragment retains very weak ARS activity.

In *S. cerevisiae*, ARSs retain function when truncated to fragments shorter than 100 bp provided the DNA fragment includes the ACS and flanking elements required for function [17,49]. To further delineate the molecular determinants of *Lk*ARS function, we constructed plasmids bearing truncated versions of 5 *Lk*ARSs (Figure 4, Additional File 2, Figure S1). Fragments bearing DNA elements sufficient for *Lk*ARS function retain their ability to maintain plasmids and generate colonies, whereas the deletion of functionally essential sequences would destroy ARS function. In all 5 cases, shortened fragments of *Lk*ARSs contained A/T sequences resembling the putative 9 bp-11 bp *Lk*ACS (Figure 3A).

**Figure 5:**
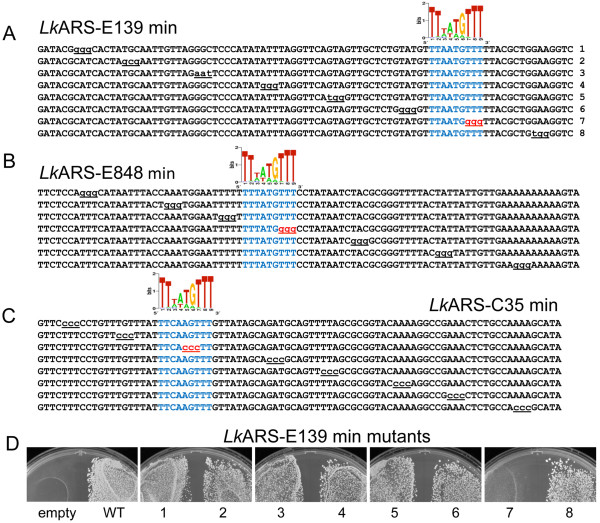
**Mutagenesis of *Lk*ARSs to identify sequences necessary for *Lk*ARS function**. (A-C) the shortest functional fragments of the three *Lk*ARSs in Figure 4 were mutated and tested for ARS function. The mutated residues are underlined. Mutations that disrupted ARS function are colored in red. The motif logos correspond to the best match of the predicted 9 bp *Lk*ACS and the relevant sequence is colored blue. (D) Representative examples of ARS function. *Lk*ARS-E139 mutant plasmids transformed into *L. kluyveri *and plated on selective media. The numbers correspond to mutant ARS fragments in (A). 'Empty' denotes the empty vector negative control, 'WT' denotes the full length *Lk*ARS-E139 positive control.

We isolated functional fragments of three of the *Lk*ARSs (*Lk*ARS-E139, *Lk*ARS-E848, and *Lk*ARS-C35) that are shorter than 100 bp and performed mutagenesis scanning experiments to identify specific sequences necessary for the ARS function of these DNA fragments (Figure 5). Using site-directed mutagenesis we systematically replaced tri-nucleotides every 8-11 bp within the functional *Lk*ARS fragments. The resulting mutants were tested for ARS function. In each of the three cases we identified a single mutant which destroyed ARS function (Figure 5).

If the putative *Lk*ACS is necessary for *Lk*ARS function, then the deleterious mutations would be located in regions that fit the best match to the *Lk*ACS motif. Conversely, we can use this data to identify the most informative ACS motif: such a motif would have better matches in all the functional mutants than in the non-functional mutants. We used the site scan mode of the program SADMAMA [50] to find in each of those 26 sequences the best match to each of the PWMs representing our candidate *L. kluyveri *ACS motifs. We then compared the score of each sequence (as determined by the best match to the PWM) against the observed functionality of that sequence. In particular, for each PWM we ranked the sequences within each of the three sets of ARS mutations according to the best PWM match score (Figure 5D). We then inspected the relative ranking, within each set, of the non-functional mutations with respect to all functional mutations.

We found that our putative *L. kluyveri *motifs of lengths 9 bp, 12 bp, 13 bp and 16 bp correctly ranked the non-functional mutations of a given ARS below all the functional mutations of that ARS. The motif of length 9 bp showed particularly good separation between the functional and non-functional scores. All other candidate *L. kluyveri *motifs did not properly rank *all *sets of mutations (Figure 5, Additional File 2, Figure S2). For this reason, we used the core 9 bp putative *Lk*ACS for further analyses.

### The selectivity of the ACS PWM models

Going back to our observation of the different levels of selectivity of the replication machinery in our three yeast species (Figure 2) we ask whether those differences are mirrored in the varying degrees of selectivity of the corresponding ACS PWM models. By selectivity of a PWM model we refer to the ability of the model to distinguish between sites generated by the model and "sites" observed in "random DNA". The results summarized in Additional File 1, Table S3 show that the ranking of the selectivity of our three species ACS PWMs is consistent with our interchangeability data: *Lk*ACS is the least while *Kl*ACS is the most selective (see Methods section for details). While this consistency is reassuring, it is difficult to draw any further conclusions from it as this PWM selectivity is a feature of the model and we have yet to establish the validity of these models. This is the subject of our next section.

As an aside we note that our definition of selectivity of a PWM is highly correlated with its information content [51] but the former is more readily interpretable as comparing the information content of PWMs of varying lengths can be quite challenging.

### The Predictive Power of ACS Motifs for ARS Function

To assess the effectiveness of our ACS models in predicting ARS functionality in their respective species we use an extended version of our ARS interchangeability data presented earlier. Specifically, for each ACS model we test how well it predicts the host species functionality (functional, non-functional, or weak) of the set of "foreign" ARSs. The latter set consists of all the ARSs that were originally screened in one of the other two species. For example, in the case of *L. kluyveri *the set of foreign ARSs consists of 401 ARSs that were screened either in *S. cerevisiae *or *K. lactis *[20,50]. Of these 401 foreign *L. kluyveri *ARSs, 299 are functional in *L. kluyveri*, 37 are weakly functional and 65 are non-functional (Additional File 1, Table S4). The prediction is specified in terms of the score of the foreign ARS' best match to the host species ACS PWM (determined by SADMAMA).

We used several correlated measures to gauge the predictive power of each species PWM model: a 2-class aROC (area under the ROC curve) that measures how well the model can distinguish between functional and non-functional (foreign) ARSs, a 3-class aROC that measures how well it can distinguish between all three functional categories (non-, weak and functional) and a measure of how well the weak ARSs are placed between the non-functional and functional ones (see Methods for details of all three measures).

All three measurements gave the same consistent answer: the predictive power of the ACS motif is highest in *K. lactis*, followed fairly closely by *S. cerevisiae *with the *L. kluyveri *ACS trailing far behind these two (Additional File 1, Table S5). Moreover, approximate 95% confidence intervals we constructed for the aROC show that the predictive power the *Lk*ACS is statistically significantly lower than that of the *Kl*ACS and the *Sc*ACS (Additional File 1, Table S5).

The rightmost column of Additional File 1, Table S5 shows that both the ACS models of *S. cerevisiae *and *K. lactis *are able to correctly resolve the weak ARSs in the sense that over 50% of the time they are correctly scored between the functional and non-functional foreign ARS. In contrast, the 9 bp *L. kluyveri *model cannot distinguish between the weak ARSs and the other two categories: it correctly places the weak ARS between the functional and non-functional ones just over 1/3 of the time (a random predictor would succeed about 1/3 of the time, see Methods for details).

The last observation as well as its generally low predictive value raise the question of whether our 9 bp *Lk*ACS model, selected based on the mutagenesis experiment, fails to capture some of the sequence elements that are crucial for a functioning *Lk*ARS. To investigate this possibility we first checked whether some of the longer putative ACS motifs offer a significantly improved predictivity. While a couple of the longer motifs do offer a slightly better predictive power, the 95% confidence intervals show that none of these differences are statistically significant and moreover these motifs are not fully consistent with the mutagenesis experiments (Additional File 1, Table S6). Among the candidate *Lk*ACS motifs that are consistent with these experiments the width 9 has the highest aROC.

### Analysis of auxiliary sequence elements

Another possible explanation for the weak predictivity of our *Lk*ACS model is that ARS activity is conferred by several sequence elements that cooperatively offer binding sites for the replication initiation machinery. This could also help explain the lack of consensus between the 9-17 bp and the group of longer putative motifs. We therefore searched for auxiliary motifs in our set of 84 native *L. kluyveri *ARSs by masking out the sites of the 9 bp ACS motif. While several of these reported motifs are statistically significant (Additional File 2, Figure S3) we did not observe any particular positional preference with respect to the location of the candidate 9 bp ACS site that was removed. Such a preference is often observed when cooperative binding takes place. In addition, these significant motifs are largely T-rich.

**Figure 6:**
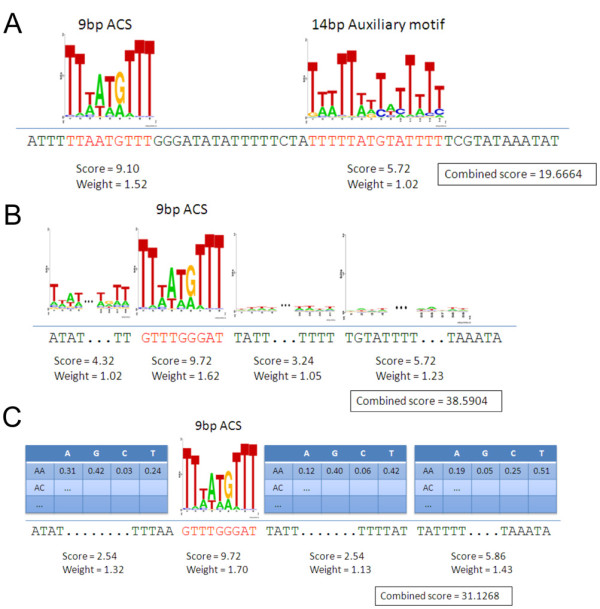
**Extended sequence models**. Graphical representation of the three linear weights models we studied that factor sequence information beyond the ACS. The paired linear model (A) is using an auxiliary motif in addition to the ACS PWM: the overall score is the weighted sum of the individual (disjoint) match scores. The contextual PWM model (B) consists of the weighted sum of the ACS match and the adjacent matches to the contextual PWMs. The latter are learned from the sites flanking the ACS sites in the alignment of the native ARSs. The Markov contextual model (C) combines the ACS match with the (log of the) Markov likelihood of the adjacent segments (normalized by an iid background model). The contextual Markov models are learned from the alignment of the native ARSs.

Nevertheless, we tested whether an augmented sequence model that combines any of these auxiliary motifs with our ACS model could offer a significantly improved predictivity of *Lk*ARS function. Specifically, we use a "paired linear model" that assigns to each sequence a score that is the maximal weighted sum of two PWM match scores: one for the putative 9 bp ACS and the other for a candidate auxiliary motif (Figure 6A). Note that the two matches cannot overlap so this optimal score is generally not the same as taking the weighted sum of the respective optimal matches of each of these two PWMs. The optimal weights are learned and evaluated for their ability to predict the functional category of each of the foreign ARSs using a 10-fold cross validation scheme (see Methods for details).

The estimated aROCs in Additional File 1, Table S7 show that combining some of the auxiliary motifs with the ACS can improve the model's predictivity. Note that the approximate 95% confidence intervals for the 2-class, as well as for the 3-class, aROC for each of the combined models heavily overlap the corresponding 95% confidence for the aROC of the ACS alone (Additional File 1, Table S5). A more powerful statistical test than simply comparing the confidence interval gives a mixed result: the improvement that the 25 bp auxiliary motif adds to the ACS model is statistically significant for the 2-class aROC but not for the 3-class aROC (for details see the section Constructing approximate confidence intervals for the cross-validation procedure in the Methods section).

Alternative approaches to utilizing extended sequence information for predicting origin locations were introduced by Breier *et al. *[52] (in *S. cerevisiae*) and more recently MacAlpine *et al *[53] (in *D. melanogaster*). In both cases the causal link between the computational predictor and the biological function is not fully understood yet both have been fairly successful at predicting origin locations in their respective species. We next describe applying variants of these methods for predicting ARS functionality in *L. kluyveri*.

### PWM contextual model

Breier *et al. *[52] showed that they can increase the power of predicting *S. cerevisiae *ARSs by extending their positional model significantly beyond the *Sc*ACS. Specifically, their model extended over 268 bp starting with a T-rich region that ends with the ACS (T-rich in itself) and is followed by an A-rich region. Interestingly, although the *Kl*ACS is very different from the *Sc*ACS, the same pattern of a T-rich region preceding the *Kl*ACS, and an A-rich region following it, emerges (Additional File 2, Figure S4). A similar pattern also emerges in L. kluyveri regardless of which specific candidate LkACS motif was used. Note that this observation can potentially explain the longer, T-rich, candidate ACS motifs, as well as the T-rich auxiliary motifs described above.

We compared the extent to which the patterns we observe in these extended profiles can improve our prediction of foreign ARS function in each of our three yeast species. For each species, we divided each of the extended sequence profiles into segments based on visual inspection, with one segment reserved for the ACS itself, and estimated a PWM for each segment. A candidate match is then scored as a weighted sum of PWM match scores, one for each segment (Figure 6B).

We again used cross validation to learn the optimal weights for each species and evaluate the corresponding predictivity of the resulting combined model (see Methods for details). The results of this analysis are presented in Additional File 1, Table S8. Using PWMs to capture the signal contained in the regions flanking the ACS (referred to below as the PWM contextual model) improves our ability to predict the foreign ARS functionality in all three species with arguably the only pronounced improvement being in *L. kluyveri *where we again used the 9-bp candidate *Lk*ACS (See Methods for details). Importantly, the improvement, offered by the contextual PWM model over the ACS-only model, in predicting the foreign ARS function in *L. kluyveri *is statistically significant: the 95% confidence interval for the difference between the (2-class) aROC of the two methods is (0.08,0.27). Similarly, the predictivity of the contextual PWM model is statistically significantly better than that offered by the combined model of the ACS and the 25 bp auxiliary motif: 95% confidence interval for the aROC is (0.0004,0.28). Similar statistically significant improvements are observed in the differences of the 3-class aROC: (0.07,0.36) and (0.03,0.18) respectively. Finally, the contextual model demonstrates a significant improvement over the ACS-only model in correctly placing the 'weak' ARSs between properly ordered functional and non-functional ARSs (Additional File 1, Table S9, rightmost column).

Unlike Breier *et al. *[52] we find that the *S. cerevisiae *PWM contextual model shows only a modest improvement over the *Sc*ACS model in predicting the function of foreign ARSs in *S. cerevisiae *and our test shows that this improvement is statistically insignificant for the given data. This may be due to the fact that at 33 bp the *Sc*ACS motif used here is significantly longer than the 17 bp ACS used by Breier *et al.*, so most of the improvement is already contained in our extended 33 bp *Sc*ACS. For *K. lactis *the improvement is marginal.

To test whether the overall span of the *L. kluyveri *contextual model (50 bp + 9 bp + 41 bp + 59 bp) can be significantly reduced, we examined two intermediate PWM contextual models between this full-length model and the one using only the 9 bp *Lk*ACS. The first added to the 9 bp ACS a 25 bp PWM on both sides and the second used the first three of the full model's four PWMs (dropping the last, 59 bp, PWM). Using the same 10-fold cross-validation scheme we found that the predictive power of these intermediate models is ordered exactly as we would expect assuming the full-length model cannot be trimmed (Additional File 1, Table S9).

Taken together our contextual PWM model provides statistically sound evidence that ARS activity in *L. kluyveri *is determined from the sequence information that is spread over a fairly large region that includes the ACS. This is quite different than the case of *K. lactis *where the 50 bp ACS is necessary and sufficient for ARS activity. Our analysis also shows that *S. cerevisiae *is much closer to *K. lactis *than to *L. kluyveri *in this regard with the 33 bp ACS predicting ARS activity with fairly high accuracy.

### Markov contextual model

Recently MacAlpine *et al *[53] showed that locations of DNA replication origins in *Drosophila *can be well predicted by relying on a support vector machine (SVM) using as features the frequencies of characteristic *k*-mers of varying length. We do not have enough data on *L. kluyveri *ARSs to apply the SVM approach here (their analysis relied on 10,000 origins). However, their analysis raises the question of whether our use of PWMs to model the segments flanking the ACS is optimal. To partly address this question we studied an intermediate model between the PWM contextual model and their SVM approach that we describe next.

We begin as in the PWM contextual model by partitioning the alignment of the native ARSs into segments, with one segment reserved for the ACS. We then learn a low order Markov chain (3^rd ^order was most commonly used) for each of the alignment segments instead of the PWM we previously used to model that segment. Consequently, each non-ACS segment is scored using its Markov chain likelihood and the score of the whole match is again the weighted sum of the scores of all the segments including the ACS segment, which still uses a PWM (Figure 6C). Using cross-validation again to estimate the performance of this approach to predicting foreign ARS functionality we found that in all three species the estimated predictive power is inferior to that of the PWM contextual model (Additional File 1, Table S10). This suggests that the sequence elements conferring replication are more constrained spatially in *L. kluyveri *than they are in *Drosophila*.

## Discussion/Conclusions

The study of eukaryotic replication origins in budding yeast has been largely limited to the well-studied *S. cerevisiae*. It has been shown that replication origins in *K. lactis*, a pre-WGD budding yeast species, are dramatically different in sequence structure than *S. cerevisiae *origins [20,40,41]. This finding suggests that other yeast species may have also evolved distinct functional paradigms for selection of DNA sequences as sites of replication initiation. In this study we have characterized origins/ARSs from another pre-WGD species - *L. kluyveri*. The ARSs used by this species, while not as different from *S. cerevisiae *as *K. lactis *ARSs, show a number of novel properties.

We used the classic ARS screen [7] to identify a large number of *Lk*ARSs (Figure 1). In addition, we tested *Sc*ARSs and *Kl*ARSs for function in *L. kluyveri *and used this information to test and improve our understanding of *Lk*ARS function. Our computational and experimental studies have identified a *Lk*ACS motif that resembles the well-studied *Sc*ACS (Figure 3A). This similarity helps explain the higher rate of common foreign ARS usage observed between *L. kluyveri *and *S. cerevisiae *than between any of them and *K. lactis *(Additional File 1, Table S4).

Despite this similarity at the ACS level, replication origins in *L. kluyveri *exhibit unique characteristics that are very different from origins in the other two yeast species. First, the sequence determinants of origin function seem to spread out over a much longer sequence. While our contextual models in *S. cerevisiae *and *K. lactis *do not show a significant improvement in predictive power over the ACS-only model, the predictive power of the contextual models in *L. kluyveri *substantially grow with the length of the context (Additional File 1, Table S9). Moreover, the difference in the predictive power of our full *L. kluyveri *PWM contextual model and the ACS-only model is statistically significant. Second, the *L. kluyveri *replication machinery exhibits a permissive behavior (Figure 2, Additional File 1, Table S4). Our PWM selectivity analysis (Additional File 1, Table S3) might explain the differences observed between the permissiveness of *L. kluyveri *and *K. lactis*. However, given that this analysis is based only on the 9 bp ACS and that the replication sequence determinants in *L. kluyveri *extend over a much longer sequence, the relatively small difference in selectivity between the *L. kluyveri *and *S. cerevisiae *ACS cannot fully explain the large difference in their acceptance rates of foreign ARSs (Additional File 1, Table S4). An alternative explanation, phrased in terms of a control system, is that the replication initiation machinery of *S. cerevisiae *and even more so of *K. lactis *have a sharp response curve as a function of the sequence composition: these machineries do not tolerate significant deviations from the optimal composition. On the other hand, the corresponding machinery of *L. kluyveri *has a much flatter response curve, in other words, it can tolerate sequences that deviate significantly from its peak response, albeit with reduced efficiency.

We have evidence that supports this flat response curve hypothesis. First, the same computational models that show very high accuracy in predicting ARS function in *S. cerevisiae *and *K. lactis *are significantly less adept in predicting ARS function in *L. kluyveri*. Second, *L. kluyveri *is far more accepting of foreign ARSs than the first two species are. Thirdly, about 22% of the foreign ARSs tested for function in *L. kluyveri *exhibit "weak" function compared with only 4% and 1% of the foreign ARSs tested, respectively, in *S. cerevisiae *and *K. lactis *(Additional File 1, Table S4). In other words, we see a statistically significant higher number of cases of much reduced, albeit functional, replication initiation in *L. kluyveri *than in the other two species (Fisher Exact Test p-values of 1.2e-8 and 3.6e-9 respectively). Note that while the first point might simply indicate our model is too crude, the last two points suggest that this may be a real biological property rather than a modeling artifact.

This behavior of *L. kluyveri *ARS function is reminiscent of the stochastic mechanism of ARS selection utilized by *S. pombe *[24,25]. However, our experiments show that *L. kluyveri *does not use an entirely stochastic mechanism for origin selection. Our scanning mutagenesis experiments have delineated specific essential sequences in each ARS tested while other similar sites are not required for function. In addition, stretches of T-rich DNA are not able to initiate DNA replication in *L. kluyveri *suggesting some level of sequence specificity. Experiments measuring replication timing show that approximately two-thirds of our *Lk*ARSs fall in zones of active replication (G. Fischer, personal communication). These findings suggest that the *Lk*ARS function mechanism is more reminiscent of *S. cerevisiae *than of *S. pombe*, yet bearing hallmarks of relaxed sequence selection requirements.

A difference in DNA sequence requirements must correspond to a difference in the protein machinery that interacts with origin DNA. The stochastic mechanism used by *S. pombe *is largely achieved by a long AT-hook domain on the N-terminus of Orp4, a subunit of ORC [23,54]. Comparison of the protein sequence of *Sc*Orc4 and *Lk*Orc4 reveals that the *L. kluyveri *version of this protein has an extended N-terminal sequence that is not present in *Sc*Orc4 (not shown). In addition, this extended N-terminal domain is not present in other related budding yeasts, even other pre-WGD species such as *Lachancea waltii*, a close relative of *L. kluyveri*. It is thus tempting to speculate that the permissive nature of *Lk*ARS function is conferred by an extended N-terminus of *Lk*Orc4 in a manner similar to *Sp*Orp4. In support of this hypothesis, the N-terminal extension is not observed in the Orc4 of *L. waltii *while recent work shows that *L. waltii *does not demonstrate the permissive features of ARS selection and instead uses a more defined ACS motif (S. Di Rienzi, K. Lindstrom, M.K. Raghuraman, B.J. Brewer, personal communication). However, the extended domain of *L. kluyveri *Orc4 does not share similarity to the AT-hook domain of *S. pombe *and we have yet to directly test its structural and functional significance. Further investigation of the molecular determinants of *L. kluyveri *ARS function will shed light on the divergence of the relationship between origin sequence and DNA replication initiation machinery.

Finally, we caution that while we consistently used a 9 bp model for the *Lk*ACS the exact length might vary. However, we note that the 9 bp is one of the few putative ACS motifs that correctly identify the essential elements of our mutagenesis experiment experiment^1 ^and it has the highest predictive power among those models. Moreover, we constructed several contextual models based on other putative ACS motifs (including length 11, 13 and 16) and none of these models had a higher predictive power than the 9 bp based model.

## Methods

### Construction of vector pIL07

As described [20]. The pIL07 plasmid was made from the pUC19 subcloning vector for the purpose of isolating ARSs. *S. cerevisiae LEU2 *gene was PCR amplified with primers containing *XbaI *sites, digested and ligated into the *XbaI *site in pUC19. *URA3 *and *S. cerevisiae CEN5 *were cloned similarly into the *EcoRI *and *HindIII *sites respectively. The *BamHI *site used for screening ARS fragments is located between the divergently transcribed *URA3 *and *LEU2 *genes. Full sequence of this vector is available upon request.

### Construction of *L. kluyveri *Genomic Libraries

Genomic DNA from the sequenced diploid FM479 strain of *L. kluyveri *was isolated using standard methods. Cells were broken using glass bead lysis and the genomic DNA was separated from mitochondrial DNA using a standard CsCl gradient protocol. Following the gradient, DNA was precipitated with 75% ethanol and resuspended in water. Prior to library construction, the DNA was digested with *MboI *and treated with Antarctic Phosphatase (New England Biolabs) to prevent the multimerization of genomic fragments. The digested insert DNA was ligated into the unique *BamHI *site in the pIL07 vector [20] using T4 DNA Ligase (New England Biolabs). The ligation reaction was purified using a PCR Purification Kit column (QIAgen) and redigested with *BamHI *to linearize all empty pIL07 molecules. The resulting reaction was used to transform chemically competent *E. coli *cells using standard methodology. In order to estimate library coverage we performed colony PCR on a 24 E.coli colonies containing plasmid clones from a set of transformation reactions. The primers used (IL325, IL326, see below) anneal to the vector sequence flanking the cloned inserts and the amplified products were analyzed by agarose gel electrophoresis to determine cloning efficiency and insert sizes. The insert sizes ranged from ~50 bp to > 2 kbp with an average length of 350 bp. Cloning efficiency ranged from 50%/reaction to 80%/reaction. Each plate of *E.coli *colonies covered an average of 75 kb of genomic DNA. Each plate was scraped separately to pool the E.coli colonies, and plasmids were extracted using the Wizard Plus SV Miniprep Kit (Promega) prior to yeast transformation.

### Screening of *L. kluyveri *Libraries for ARS Activity

The plasmid libraries were used to transform a *ura3 *auxotrophic *L. kluyveri *strain FM628 using a standard Lithium Acetate protocol [55] and plated on medium lacking uracil to select for ARS function. One yeast colony per library transformation was re-streaked onto fresh plates and subsequently grown in culture medium lacking uracil to enrich for the ARS bearing plasmid. The plasmids were isolated from yeast using a modified DNA extraction protocol. 2 mL of culture was pelleted and resuspended in 500 μL of a buffer consisting of 1 M sorbitol, 0.1 M EDTA, 1 μL/mL β-mercaptoethanol, and 0.5 mg/mL Yeast Lytic Enzyme (VWR, catalog # IC360951). The suspension was incubated at 37 degrees for 1 hour. The treated cells were pelleted and processed using the Wizard Plus SV Miniprep kit (Promega). 5 μL of the resulting eluate was used to transform *E. coli *and transformants were miniprepped to isolate the ARS-bearing plasmid. A small sample of each plasmid was used to re-transform FM628 to confirm ARS function. Confirmed ARS plasmids were sequenced using primers IL325 (5'-GCCAAACAACCAATTACTTGTTGAGA-3') and IL326 (5'-TTCGTTGCTTGTCTTCCCTAGTTTC-3') from both ends of the ARS fragment. All *Lk*ARSs identified are listed in Additional File 3.

### Cloning, Truncation and Mutagenesis of ARS sequences

As described [20]. To clone and test predicted regions of DNA for ARS activity, primers containing *BamHI *and *BglII *cloning sites were designed to anneal to the relevant region. PCR amplified DNA was cloned into pIL07 and/or pRS406 and confirmed by sequencing. Several clones of each predicted region were used to transform MW98-8C to test for ARS function. ARS fragments were truncated by amplifying and cloning smaller fragments of the ARS region. Site-directed mutagenesis was performed using a fusion-PCR mutagenesis method [56]. The DNA region to be mutagenized was PCR amplified in two separate fragments, which overlap by 50 - 60 base pairs. The overlap primers contained the desired mutation. After the initial PCR, the two fragments were purified separately and used together in another PCR reaction without any template DNA. The overlapping regions in the two DNA fragments acted as primers for each other and PCR produced a final molecule which contained the entire DNA fragment including the mutation of interest. This fragment was then cloned into the vector and sequenced as above.

### Analysis of ARS interchangeability data

We categorized the functionality of each *Lk*ARS in *S. cerevisiae *and *K. lactis *as 'no', 'weak', or 'yes' according to a visual inspection of its ARS functionality in the respective host species. *Lk*ARS function in other species is listed in Additional File 3.

We assessed the statistical significance of the number of *Lk*ARSs that function in *both *of the other two species using a hypergeometric test. More precisely, we conducted 3 tests one for each of the three possible interpretations of a weakly functional ARS: ignoring any ARS that is classified as 'weak' in either one of the two host species, classifying a 'weak' ARS as 'yes', and classifying a 'weak' ARS as 'no'.

The hypergeometric test measures the level of surprise of the size of the intersection between two sets, in our case the set of *Lk*ARSs that function in *S. cerevisiae *and the set of *Lk*ARSs that functions in *K. lactis*. Specifically our model is that we sample without replacement *n *balls (the *K. lactis *'yes' *Lk*ARSs) out of an urn with *m *black balls (the *S. cerevisiae *'yes' *Lk*ARSs) and N-m white balls (the *S. cerevisiae *'no' *Lk*ARSs). Let *k *be the number of sampled black balls, then *k*, which is the number of *Lk*ARSs that function in both species, is statistically significant if the probability of observing in our sample *k *or more black balls is below 0.05. This probability is given by the hypergeometric distribution function.

We repeated this test for evaluating the size of the set of *Sc*ARSs that function in both *K. lactis *and *L. kluyveri *and the size of the set of *Kl*ARSs that function in both *S. cerevisiae *and *L. kluyveri*. We evaluated the p-values for the three different possible classifications of weak functionality for all three sets of foreign ARSs. Only the intersection of *Kl*ARSs that function in both *S. cerevisiae *and *L. Kluyveri *was statistically significant and in this case it was significant *regardless *of how we classify weak functionality: 0.002 (weak = functional), 0.006 (ignoring weak), 0.018 (weak = non-functional).

### Identification of ACS position weight matrix (PWM)

The *L. kluyveri *genome we downloaded and analyzed is the Genolevures 2008 09 version of NRRL Y-12651 (aka CBS3082) project accession AACE00000000 http://www.genolevures.org/download.html#sakl.

We used the given annotations to generate a file of *L. kluyveri *intergenic sequences by filtering out all sequences with feature type 'gene' or 'LTR', or 'gap'.

When searching for the *L. kluyveri *ACS GIMSAN was applied to the set of 84 *L. kluyveri *ARSs that were uniquely mapped to the *L. kluyveri *genome with the aforementioned intergenic file and the additional parameters:

--w = 7,8,9,10,11,12,13,14,15,16,17,25,30,40,50 --oops --t = 200 --L = 200 --nullset = 50 --markov = 4

The "*S. cerevisiae *intergenic file" was generated by removing all sequences with feature type 'gene' or 'LTR', or 'gap' from the October 2003 GenBank version of the *S. cerevisiae *genome.

To define the *S. cerevisiae *ACS motif GIMSAN was applied to a list of 324 verified *S. cerevisiae *ARSs that is largely based on the list of OriDB confirmed ARSs and that was compiled as explained in [20]. In addition to the aforementioned *S. cerevisiae *intergenic file GIMSAN was given the following parameters

--w = 11,17 --oops --t = 200 --L = 200 --nullset = 0 --markov = 4

### Assessing the selectivity of a PWM

We define the selectivity of a motif as its ability to distinguish between sites generated according to the motif model (PWM in this case) and sites occurring in "random DNA". Specifically, if we imagine we have a list of "real sites" of length l that are generated by the model and a null generated list of N l-mers we can ask how many null sites vs. real sites score above the threshold which we vary. This is a special case of an ROC (receiver operating characteristic) curve and as such it is summarized by the area under the curve (aROC). A maximally selective PWM will have an aROC of 1 (all real sites score higher than all null sites) whereas a non-selective PWM will have an aROC of 0.5 (a real site is equally likely to score higher or lower than a null site.

For each species putative ACS motif (we used the 9 bp *Lk*ACS, the 33 bp *Sc*ACS and the 50 bp *Kl*ACS) we compiled a PWM from all the sites selected by our motif finder when applied to the set of the species' native ARSs and added a pseudocount of 0.01. Using each of these PWMs at a time we sampled 200 sites using the canonical independent column assumption. We then added to the right and left of each sampled site flanks of length 4 bp sampled uniformly from the corresponding set of flanks of the same putative motif sites that are used to define the PWM^2^.

We then generated 200,000 "null sites" by sampling a sequence of length L (where L = 100,000 + width of motif - 1) from a background 4th order Markov chain trained on the species' set of intergenic sequences. The null sites were defined by the list of all the words in this string and its reverse complement. We then scored the 200 sampled "real sites" as well as these 200,000 sampled "null sites" using an LLR (log-likelihood ratio) score: a site score is the log of the ratio of the likelihood of the site under the PWM model over the likelihood of the site under the background Markov model. The slight twist we introduced here is to average the background likelihood over the two "strands", i.e., over the site and its reverse complement.

We then used the canonical measure of aROC to gauge how well the PWM distinguishes between the null and real sites.

### Estimating the predictive power of an ACS PWM

Using each host species ACS PWM we assign each foreign ARS a score that is the score of the best match to the ACS (assigned by SADMAMA). We then rank each foreign ARS according to its score and evaluate, using the standard measure of aROC, how well this ranking agrees with the ARS functionality of these sequences in the host species. A perfectly predictive classifier (or PWM in this case) would give an aROC value of 1 while a random classifier would give an aROC of ~0.5. When using the aROC we can only define 2 classes so we use only the functional and non-functional set of ARSs in this evaluation, leaving out all weak ones.

To utilize the set of weak ARSs we note that the aROC has an equivalent probabilistic formulation. Namely, if you imagine randomly drawing one sequence from the functional and one from the non-functional set of foreign ARSs, then the aROC is the probability that the (classifier) score of the functional sequence will be higher than the score of the non-functional sequence. One advantage of this latter formulation is that it suggests an obvious generalization for more than 2 classes. For example, when we have 3 linearly ordered classes, as in our example (non-functional < weak < functional), we can define the generalized aROC as the probability that the scores of a randomly drawn triplet of sequences, one from each class, will be ordered correctly: the score of the non-functional sequence is the smallest and the score of the functional sequence is the highest. Note that while a perfect classifier would still have a generalized aROC of 1, the generalized aROC of a random classifier on 3 classes would be roughly 1/6 ~ 0.167 as there are 6 different permutations or ways to order the scores of the 3 sequences.

The summary of our analysis of the 3-class, or generalized, aROC is presented in Additional File 1, Table S5. The ranking of the predictive power of the ACS PWMs of the 3 species is the same as for the 2-class, or standard, aROC. The confidence interval for the predictive power of the *Kl*ACS is unusually wide since there is only one foreign *Kl*ARS that is weakly functional.

Combining the information from the 3-class and the 2-class aROC allows us to gauge the predictive power of our models on a finer scale than we can when using the standard 2-class aROC. For example, we can test whether the scores our models assign to the weak ARSs are correctly placed between the functional and the non-functional foreign ARSs. More precisely, we compute the probability that a randomly drawn weak foreign ARS is correctly placed between a randomly drawn pair of functional and non-functional foreign ARSs, *conditioned *on that pair being correctly ordered: the score of the non-functional foreign ARS is smaller than the score of the functional one. This conditional probability is given by the ratio of the 3-class aROC to the 2-class aROC. As there are 3 possible outcomes for placing the weak ARS score relative to the selected ordered pair, a ratio of 1/3 is essentially random: the model is not able to resolve the more subtle differences between weak ARSs and functional/non-functional ones.

### Confidence intervals for aROC of predicting functionality of foreign ARSs

To account for the random effects in evaluating the aROC we used bootstrap to construct approximate 95% confidence as described next. Each foreign ARS is assigned a score, corresponding to the best match to the host species ACS, as well as a label describing its ARS functionality in the host species: functional, non-functional, or weakly functional. Thus, for each host species we have 3 lists of foreign ARS sequence scores: functional, non-functional and weak. We sample with replacement each of the three lists separately to generate 10,000 bootstrapped score lists of the same size as the original. We then compute the aROC for the bootstrapped functional and non-functional lists and compute the 3-class aROC using all three bootstrapped lists. This provides us with an empirical sample of 10,000 aROCs from which we generate approximate confidence intervals as described next.

When the host species was *L. kluyveri *the empirical sample was well approximated by a normal distribution as verified using a QQ plot (data not shown). Therefore we constructed normal 95% confidence intervals based on the standard deviation estimated from the empirical sample.

When *S. cerevisiae *and *K. lactis *are host species, the normal assumption is not well supported in the empirical sample. This is probably due to the smaller sample sizes as well as the proximity of the point estimator of the aROC to the maximal possible score of 1. Therefore in these two cases we used the 0.025 and 0.975 quantiles of the empirical distribution as a proxy for the 95% confidence intervals. To validate this approach we compared the quantiles based intervals with the normal derived intervals in the case of *L. kluyveri *and found good agreement between the two.

### Searching for an auxiliary motif

GIMSAN was applied to the set of 84 native *Lk*ARSs after the length 9 bp motif site was masked out in each sequence. We used the *L. kluyveri *intergenic background file mentioned above and the following additional parameters:

--w = 6,7,8,9,10,11,14,17,25 --oops --t = 200 --L = 200 --nullset = 50 --markov = 4

--w = 6,7,8,9,10,11,14,17,25 --zoops = 0.2 --t = 200 --L = 200 --nullset = 50 --markov = 4

### Evaluating the paired linear model

We considered paired linear models defined by the 9 bp putative *L. kluyveri *ACS PWM and an auxiliary PWM. The model had 2 parameters which are the positive weights assigned to each of the two PWMs. Given the weights and a match to each of the PWMs the score is the weighted sum of the standard scores assigned to each PWM match. The score of a sequence is defined as the maximal weighted match scores subject to the constraint that the matches cannot overlap. As the best matches to each of the PWM can potentially overlap in any given sequence, this sequence score is not necessarily equal to the weighted sum of these two best matches.

The three auxiliary motifs we considered were selected based on the fact that two of them were assigned by GIMSAN the best overall p-values: the 14 bp motif found using a ZOOPS model and a 25 bp motif found using an OOPS model. In addition we tested a 6 bp motif reported by GIMSAN using a ZOOPS model (all 3 motifs can be inspected in Additional File 2, Figure S3).

Given a training set of ARSs for which we know the labels ('yes', 'no', or 'weak') we define the optimal pair of weights as the one that will maximize either the 2-class (in which case we ignore all 'weak' ARSs) or 3-class aROC depending on which one we are trying to optimize. The optimization is achieved using the general Powell minimization^3 ^method implemented in the Python Scipy package.

At the core of the optimization is the function that computes the score of each sequence given the current value of the pair of weights. In principle, computing this score for a given sequence involves considering every pair of sites in the sequence, one per each PWM and taking the maximum of all the corresponding weighted sums. However, we can rank the matches to each PWM and use those ranks to identify each pair of matches with a point in the 2-d integer lattice.

It is easy to see that ignoring the non-overlap constraint the maximal weighted sum will always coincide with the (1,1) point in the lattice, that is the best match to each PWM (recall the weights are positive). However, in general this point as well as others on this lattice might not be feasible due to overlap between the corresponding sequence matches. It is not difficult to see that in this general case the maximum can only be attained on the maximal lattice points among the set of feasible lattice points. Finding the latter points is something that can readily be done in a preprocessing step by going over the 2 lists of ranked matches, one for each PWM. This preprocessing significantly reduces the amount of computation required for evaluating the aROC associated with each pair of weights.

To evaluate the predictive power of our model we use cross-validation. We randomly partition the set of *L. kluyveri *foreign ARSs into n folds (we used *n *= 10 here), subject to the constraint that the classes proportion in each fold would be essentially constant. We then sequentially leave out one fold and use the remaining *n*-1 folds as a training set to find the pair of optimal weights as described above. We then use these weights to assign scores to the ARSs in the left out fold and evaluate the aROC (2-class or 3-class depending on the corresponding training target function). We next average the aROC over the *n *left out folds. Finally, we repeat this entire process 1,000 times, randomly partitioning the ARSs into *n *folds each time and report the average of the *n*-fold averaged aROC.

### Constructing approximate confidence intervals for the cross-validation procedure

In this work we used cross-validation on a number of occasions to estimate a model's aROC. As usual with such point estimates there is randomness in its exact value. In this case, the average aROC depends on the arbitrary assignment of the sequences into the *n *folds. It also depends on the set of foreign ARSs we happened to isolate. To control for these two sources of random fluctuations we constructed approximate confidence intervals using the following procedure.

We randomly partition the data into *n *folds using the same *n *that was used in the original cross-validation scheme, preserving the (2 or 3) functional classes proportions in each fold. We then bootstrap each fold separately by sampling with replacement the sequences in each class of that fold. This procedure leaves us with a bootstrapped set of *n *folds that are disjoint (although a sequence might be found more than once within a fold). We apply the original "leave out one fold at a time" evaluation scheme to this bootstrapped set of folds to obtain an aROC value. Repeating this procedure 1,000 times we generate a bootstrapped empirical distribution of the aROC. Our reported approximate 95% confidence interval is estimated from the 0.025 and 0.975 quantiles of this empirical distribution.

A variant of this procedure allows us to determine whether one method for predicting ARS function is statistically significantly better than another method. Specifically, we evaluate the difference between the two methods' aROC on each bootstrapped sample generated as above. If the 95% confidence interval of the difference lies entirely to the right/left of 0 then we say the first/second method is significantly better. The 95% confidence intervals are constructed using the normal method if that normal assumption is supported by the Lilliefors test (at the standard 5% level). Otherwise, the 0.025 and 0.975 sample quantiles are used to define the approximate 95% confidence interval.

### PWM contextual model

The alignment of the native ARSs in each species (Additional File 2, Figure S4) was visually partitioned into 3-4 segments one of which was reserved for the ACS. A PWM was learned from each segment (with the ACS segment yielding back its own PWM).

For each scanned sequence (a foreign ARS) the top 50 matches of the ACS PWM were found by SADMAMA (-pwmPC 0.01 -m 4 both_strands -siteNullScore avg_strands). A python script was written to parse the SADMAMA output and add to it the weighted scores of the neighboring segments using the contextual PWMs mentioned above. A pseudo count of 0 was used for each contextual PWM and the background model for the LLR score was a 0-th order Markov chain learned from the host intergenic sequences.

Given a training set, the weights are optimized for the 2-class aROC (or the 3-class aROC depending on the optimized target). The training sets are determined through a cross-validation scheme applied to the set of foreign ARSs. Specifically, for each species we divided its set of foreign ARSs into *n *folds (*n *= 10 for *S. cerevisiae *and *L. kluyveri*, n = 7 for *K. lactis*). Leaving out one fold at a time, we used *n*-1 of the *n *folds to learn a set of computationally optimal segment weights and used those to calculate the aROC on the left-out fold and finally report the average aROC over the *n *cycles. The 95% confidence intervals for the aROC as well as for the difference between the aROCs of alternative methods were estimated using bootstrap as described above.

We also examined the effects of allowing some flexibility at the seams between the PWMs. Specifically; we allowed some slack, a small gap or overlap, between the end of the current segment and the start of the next one. The slack in the offset from the ACS PWM was no more than *k *times the number of segments between the relevant segment and the ACS (*k *= 0,1,2 were considered). Adding this flexibility did not seem to improve the model's prediction power so all the results reported below are for the rigid case (*k *= 0).

The *S. cerevisiae *segments that were tested are (see Additional File 2, Figure S4, left pane):

• 1-100 (T-rich region); 101-133 (ACS); 134-216 (A-rich region)

The *K. lactis *segments that were tested are (see Additional File 2, Figure S4, right pane):

• 51-100 (T-rich region); 101-150 (ACS); 151-200 (A-rich region)

The *L. kluyveri *segments that were tested are (see Additional File 2, Figure S4, middle pane):

• 51-100 (T-rich region); 101-109 (ACS); 110-150 (AT-rich region); 151-209 (A-rich region)

We also tested two "shorter" *L. kluyveri *contextual models, one using the 9 bp ACS together with a pair of flanking segments of length 25 bp, and another using the first 3 segments of the 4-segments *L. kluyveri *model described above.

### Markov contextual model

The models that were tested were based on the same segmentation used in the PWM contextual model above.

## Authors' contributions

Conceived and designed the experiments: IL, MI, BKT, UK. Performed the experiments: IL, KL, SCCC, LY, AC, LH, EC, GK, HP, JB. Analyzed the data: IL ET BKT UK. Wrote the paper: IL, MI, BKT, UK. All authors read and approved the final manuscript.

## Endnotes

^1^As did the extended *L. kluyveri *PWM contextual model based on this 9 bp ACS.

^2^These flanks influence the sites scores as will become clear below.

^3^We could have used a 1-dimensional optimization here but the code was written for a more general case allowing more than one auxiliary PWM.

## Supplementary Material

Additional file 1 **Supplementary Tables**. The supplementary tables associated with this study. Click here for file

Additional file 2 **Supplementary Figures**. The supplementary figures associated with this study. **Figure S1**. Additional *Lk*ARS truncation experiments. *Lk*ARS-E143 (A) and *Lk*ARS-C1177 (B) were truncated to narrow down functional regions. Black boxes represent functional *Lk*ARS fragments, red boxes represent non-functional fragments. The extent of the truncation in basepairs is indicated on the left of the graphics (L = truncated from the left, R = truncated from the right). The length of the original full-length fragment isolated from the screen is indicated next to the first fragment from the top. **Figure S2**. The reduced predictive power of the 11 bp *Lk*ACS. As in Figure 5, but highlighted with the best match of the 11 bp LkACS motif. This motif fails to properly identify the essential region of *Lk*ARS-C35. **Figure S3**. *L. kluyveri *auxiliary motifs. The 6 bp motif (GIMSAN p-value 0.036) appeared in 29 of the 84 *Lk*ARSs, the 14 bp motif (GIMSAN p-value 0.001) in 53 of the *Lk*ARSs and the 25 bp motif (GIMSAN p-value 0.0014) in all the sequences (using the OOPS model). **Figure S4**. Nucleotide distributions surrounding functionally relevant ACS motifs in *Sc*ARSs (A), *Lk*ARSs (B), and *Kl*ARSs (C). Click here for file

Additional file 3 **ARSs used in this study**. A list of coordinates and functional information of the *Lk*ARSs used in this study. Click here for file
