# Supplementary material for: Novel features of ARS selection in budding yeast Lachancea kluyveri
Source: BMC Genomics. 2011 Dec 28;12:633. doi: 10.1186/1471-2164-12-633 (PMC3306766; doi:10.1186/1471-2164-12-633)
Supplement: Additional file 1 — Supplementary Tables. The supplementary tables associated with this study. [file 1471-2164-12-633-S1.DOC]

­­­

| **Functional and Weak (%)** | **Host** | | |
| --- | --- | --- | --- |
| **Donor** | *S.cerevisiae* | *L.kluyveri* | *K.lactis* |
| *S.cerevisiae* |  | 94.9 | 5.1 |
| *L.kluyveri* | 41.7 |  | 7.2 |
| *K.lactis* | 16.9 | 79.7 |  |
| **Functional (%)** | **Host** | | |
| **Donor** | *S.cerevisiae* | *L.kluyveri* | *K.lactis* |
| *S.cerevisiae* |  | 94.9 | 5.1 |
| *L.kluyveri* | 39.3 |  | 6.0 |
| *K.lactis* | 12.2 | 52.0 |  |

**Table S1: Percentage of foreign ARS functionality.** Each entry specifies the percentage of ARSs native to the species specified by the row that are functional in the host species specified by the column. In the top half functional and weak ARSs are lumped together whereas in the lower half only the functional ARSs are considered.

| **Donor** |  | | | | |
| --- | --- | --- | --- | --- | --- |
| ***L.kluyveri*** | **Host** | ***S.cerevisiae*** | | | |
|  | ***K.lactis*** |  | **Functional** | **Non-functional** | **Weak** |
|  |  | **Functional** | **3** | **2** | **0** |
|  |  | **Non-functional** | **29** | **46** | **2** |
|  |  | **Weak** | **0** | **1** | **0** |
| ***S.cerevisiae*** | **Host** | ***K.lactis*** | | | |
|  | ***L.kluyveri*** |  | **Functional** | **Non-functional** | **Weak** |
|  |  | **Functional** | **2** | **35** | **0** |
|  |  | **Non-functional** | **0** | **2** | **0** |
|  |  | **Weak** | **0** | **0** | **0** |
| ***K.lactis*** | **Host** | ***L.kluyveri*** | | | |
|  | ***S.cerevisiae*** |  | **Functional** | **Non-functional** | **Weak** |
|  |  | **Functional** | **14** | **0** | **4** |
|  |  | **Non-functional** | **60** | **30** | **33** |
|  |  | **Weak** | **3** | **0** | **4** |

**Table S2: Cross-tabulation of foreign ARS functionality in host species by donor and hosts.** A further breakdown of host to donor foreign ARS functionality.

|  | **50bp *Kl*ACS** | **33bp *Sc*ACS** | **9bp *Lk*ACS** | **11bp *Lk*ACS** | **16bp *Lk*ACS** |
| --- | --- | --- | --- | --- | --- |
| **1-aROC** | 1.30E-05 | 1.70E-04 | 4.90E-04 | 3.70E-04 | 3.20E-04 |

**Table S3: Selectivity of ACS PWM.** The selectivity of a PWM is defined here as its ability to differentiate between sites generated by the PWM model and those generated by a null model (a 4th order Markov chain trained on the corresponding species intergenic sequences was used here). A perfect selectivity corresponds to 1-aROC of 0 and the smaller that number is the better the selectivity. For more details see “Assessing the selectivity of a PWM” in the Methods section.

| Host species | Functional | Non-functional | Weak |
| --- | --- | --- | --- |
| *L. kluyveri* | 299 | 65 | 37 |
| *S. cerevisiae* | 51 | 172 | 9 |
| *K. lactis* | 7 | 114 | 1 |

**Table S4: Foreign ARS functionality in host species.** The table provides the summary statistics of the foreign ARS functionality broken down by host species. Note that a foreign ARS is defined as one originally screened in another species. For example, a segment of *L. kluyveri* DNA that was first screened as an active ARS in *S. cerevisiae* and only tested later for ARS function in *L. kluyveri* is considered a foreign *L. kluyveri* ARS.

|  |  | **aROC** | | **Generalized (3-class) aROC** | | **3-class over 2-class aROC** |
| --- | --- | --- | --- | --- | --- | --- |
| **Host species** | **ACS model** | **Estimator** | **~95% CI** | **Estimator** | **~95% CI** |
| *L. kluyveri* | 9bp PWM | 0.676 | (0.598 , 0.754) | 0.249 | (0.180 , 0.318) | 0.368 |
| *S. cerevisiae* | 33bp PWM | 0.899 | (0.842 , 0.948) | 0.492 | (0.372 , 0.608) | 0.547 |
| *K. lactis* | 50bp PWM | 0.974 | (0.932 , 1.000) | 0.569* | (0.143 , 0.857) | 0.584 |

**Table S5: The predictive power of the ACS PWM model.** The power of the putative ACS PWM deduced from the set of native ARSs to predict foreign ARS functionality in the respective host species as measured by aROC (see Methods for details). The last column measures the consistency of the “weak” ARS classification with the PWM score: is its score correctly ranked between the scores of a functional and a non-functional ARS; here a ratio of 1/3 is essentially random (for more details see “Estimating the predictive power of an ACS PWM” and “Confidence intervals for aROC of predicting functionality of foreign ARSs” in the Methods section).

* Note: This estimated aROC is based on only 1 weak foreign *K. lactis* ARS.

|  | **aROC** | | **Generalized (3-class) aROC** | | **3-class over 2-class aROC** |
| --- | --- | --- | --- | --- | --- |
| **Putative ACS PWM** | **Estimator** | **~95% CI** | **Estimator** | **~95% CI** |
| 9bp | 0.676 | (0.598 , 0.754) | 0.249 | (0.180 , 0.318) | 0.368 |
| 10bp | 0.687 | (0.611 , 0.762) | 0.275 | (0.206 , 0.344) | 0.400 |
| 11bp | 0.694 | (0.620 , 0.768) | 0.290 | (0.215 , 0.364) | 0.418 |
| 12bp | 0.674 | (0.597 , 0.750) | 0.295 | (0.218 , 0.371) | 0.438 |
| 13bp | 0.647 | (0.566 , 0.727) | 0.279 | (0.207 , 0.351) | 0.431 |
| 14bp | 0.640 | (0.560 , 0.721) | 0.283 | (0.213 , 0.353) | 0.442 |
| 15bp | 0.622 | (0.542 , 0.703) | 0.266 | (0.196 , 0.335) | 0.428 |
| 16bp | 0.656 | (0.579 , 0.732) | 0.261 | (0.189 , 0.332) | 0.398 |
| 17bp | 0.614 | (0.532 , 0.696) | 0.255 | (0.183 , 0.326) | 0.415 |
| 25bp | 0.701 | (0.634 , 0.769) | 0.301 | (0.228 , 0.375) | 0.429 |
| 30bp | 0.643 | (0.572 , 0.713) | 0.250 | (0.185 , 0.316) | 0.389 |
| 40bp | 0.693 | (0.624 , 0.761) | 0.295 | (0.231 , 0.359) | 0.426 |
| 50bp | 0.647 | (0.575 , 0.719) | 0.286 | (0.225 , 0.347). | 0.442 |

**Table S6: The predictive power of putative *Lk*ACS PWM models of different widths.** The predictive power was assessed in terms of aROC based on the set of foreign ARSs (for more details see “Estimating the predictive power of an ACS PWM” and “Confidence intervals for aROC of predicting functionality of foreign ARSs” in the Methods section).

|  | **aROC** | | **Generalized (3-class) aROC** | |
| --- | --- | --- | --- | --- |
| **Auxiliary motif** | **Estimator** | **~95% CI** | **Estimator** | **~95% CI** |
| 6bp PWM | 0.681 | (0.561 , 0.741) | 0.234 | (0.136 , 0.340) |
| 14bp PWM | 0.711 | (0.614 , 0.779) | 0.306 | (0.187 , 0.387) |
| 25bp PWM | 0.746 | (0.660 , 0.801) | 0.330 | (0.215 , 0.410) |

**Table S7: The predictive power of the paired linear model: ACS plus auxiliary motif.** The model is constructed to predict ARS functionality in *L. kluyveri* and is parameterized and assessed using 10-fold cross-validation applied to the set of *L. kluyveri* foreign ARSs. Predictivity is measured in terms of the aROC averaged over the 10 folds (for details see “Evaluating the paired linear model” and “Constructing approximate confidence intervals for the cross-validation procedure” in the Methods section).

|  |  |  |  | **aROC** | |
| --- | --- | --- | --- | --- | --- |
| **Host species** | **ACS PWM** | **# of contextual PWMs** | **Weights** | **Estimator** | **~95% CI** |
| *L. kluyveri* | 9bp | 4 | Optimized | 0.828 | (0.742 , 0.889) |
| *S. cerevisiae* | 33bp | 3 | Optimized | 0.916 | (0.848 , 0.960) |
| *K. lactis* | 50bp | 3 | Optimized | 0.983 | (0.920 , 1.000) |

**Table S8: The predictive power of the contextual PWM model.** The model is constructed to predict ARS functionality in the respective host species. We tried two approaches to parameterize the model. In the first, the PWM weights are optimized and the model is assessed using cross-validation applied to the respective set of foreign ARSs. In the second, we used uniform PWM weights. When using uniform weights the aROC for the *L. kluyveri* contextual model is statistically significantly higher than when using only the ACS PWM model (Supplementary Table 5). When optimizing the weights of the contextual model all 3 species improve on the ACS PWM model, however, only marginally so for *S. cerevisiae* and *K. lactis* folds (for details see “PWM contextual model” and “Constructing approximate confidence intervals for the cross-validation procedure” in the Methods section).

|  | **aROC (2-class)** | | **Generalized (3-class) aROC** | | **3-class over 2-class aROC** |
| --- | --- | --- | --- | --- | --- |
| **Model type** | **Estimator** | **~95% CI** | **Estimator** | **~95% CI** |
| **ACS alone** | **0.677** | **(0.619 , 0.764)** | **0.250** | **(0.187 , 0.322)** | **0.369** |
| ACS plus 25bp auxiliary PWM | 0.746 | (0.660 , 0.801) | 0.329 | (0.215 , 0.410) | 0.441 |
| **PWM contextual** | **0.828** | **(0.742 , 0.889)** | **0.441** | **(0.292 , 0.531)** | **0.540** |
| PWM contextual (ACS +/-25bp) | 0.722 |  | 0.337 |  |  |
| PWM contextual (ACS -50+41bp) | 0.775 |  | 0.372 |  |  |
| Markov model | 0.757 |  |  |  |  |

**Table S9: Summary of the predictive power of our *L. kluyveri* ARS models.** All models are constructed on the set of native *Lk*ARSs and tested on the set of *L. kluyveri* foreign ARSs. The predictivity is measured by the model’s aROC. All models are based on the same putative 9bp ACS PWM. In bold face are the best and worst predictors. Importantly, for these two models the difference in both the 3-class and the 2-class aROC are statistically significant: the 95% confidence intervals for these differences are entirely to the right of 0 (for further details see the section Analysis of auxiliary sequence elements).

| **Host species** | **ACS model** | **Number of segments** | **aROC Estimator** |
| --- | --- | --- | --- |
| *L. kluyveri* | 9bp PWM | 4 | 0.757 |
| *S. cerevisiae* | 33bp PWM | 3 | 0.898 |
| *K. lactis* | 50bp PWM | 3 | 0.974 |

**Table S10: The predictive power of the Markov contextual model.** The model is constructed to predict ARS functionality in the respective host species and its predictivity is measured by aROC when applied to predicting the functionality of the respective set of foreign ARSs (see Methods for details). For all 3 species the estimated aROC is lower than the corresponding estimated aROC using the contextual PWM model.
