# Supplementary material for: Novel features of ARS selection in budding yeast Lachancea kluyveri
Source: BMC Genomics. 2011 Dec 28;12:633. doi: 10.1186/1471-2164-12-633 (PMC3306766; doi:10.1186/1471-2164-12-633)
Supplement: Additional file 2 — Supplementary Figures. The supplementary figures associated with this study. Figure S1. Additional LkARS truncation experiments. LkARS-E143 (A) and LkARS-C1177 (B) were truncated to narrow down functional regions. Black boxes represent functional LkARS fragments, red boxes represent non-functional fragments. The extent of the truncation in basepairs is indicated on the left of the graphics (L = truncated from the left, R = truncated from the right). The length of the original full-length fragment isolated from the screen is indicated next to the first fragment from the top. Figure S2. The reduced predictive power of the 11 bp LkACS. As in Figure 5, but highlighted with the best match of the 11 bp LkACS motif. This motif fails to properly identify the essential region of LkARS-C35. Figure S3. L. kluyveri auxiliary motifs. The 6 bp motif (GIMSAN p-value 0.036) appeared in 29 of the 84 LkARSs, the 14 bp motif (GIMSAN p-value 0.001) in 53 of the LkARSs and the 25 bp motif (GIMSAN p-value 0.0014) in all the sequences (using the OOPS model). Figure S4. Nucleotide distributions surrounding functionally relevant ACS motifs in ScARSs (A), LkARSs (B), and KlARSs (C). [file 1471-2164-12-633-S2.PDF]

Figure S1

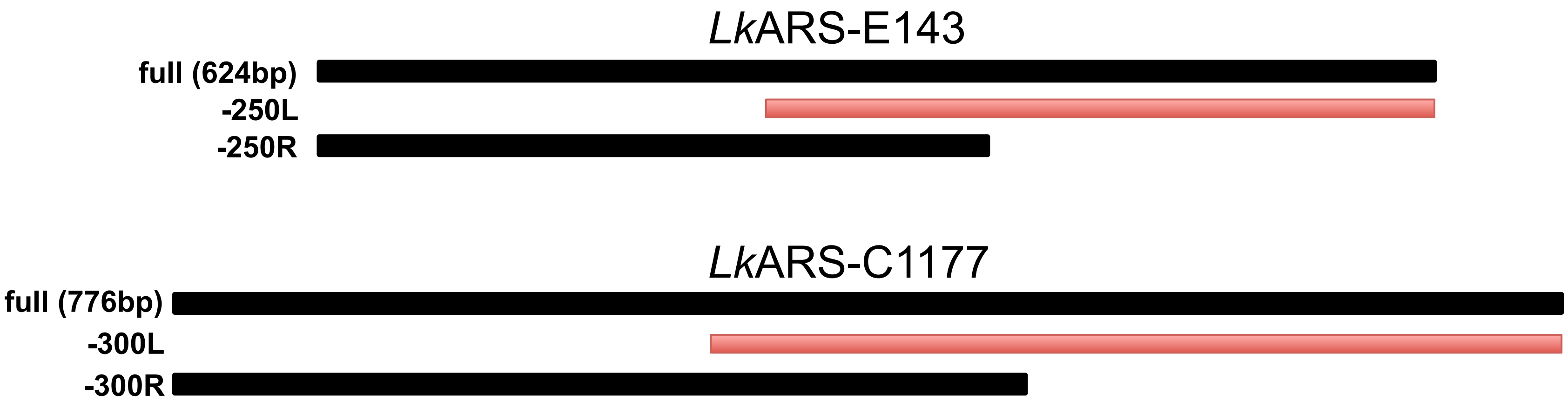
LkARS-E143
full (624bp)
-250L
-250R
LkARS-C1177
full (776bp)
-300L
-300R

Figure S2

*Lk*ARS-E139 min

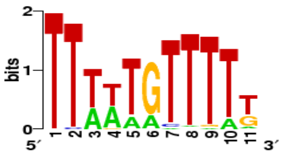

GATACGgggCACTATGCAATTGTTAGGGCTCCCATATATTTAGGTTCAGTAGTTGCTCTGTATGTTTAATGTTTTTACGCTGGAAGGTC
GATACGCATCACTAgcgAATTGTTAGGGCTCCCATATATTTAGGTTCAGTAGTTGCTCTGTATGTTTAATGTTTTTACGCTGGAAGGTC
GATACGCATCACTATGCAATTGTTAGaatTCCCATATATTTAGGTTCAGTAGTTGCTCTGTATGTTTAATGTTTTTACGCTGGAAGGTC
GATACGCATCACTATGCAATTGTTAGGGCTCCCATATgggTAGGTTCAGTAGTTGCTCTGTATGTTTAATGTTTTTACGCTGGAAGGTC
GATACGCATCACTATGCAATTGTTAGGGCTCCCATATATTTAGGTTCAtggGTTGCTCTGTATGTTTAATGTTTTTACGCTGGAAGGTC
GATACGCATCACTATGCAATTGTTAGGGCTCCCATATATTTAGGTTCAGTAGTTGCTCTGgggGTTTAATGTTTTTACGCTGGAAGGTC
GATACGCATCACTATGCAATTGTTAGGGCTCCCATATATTTAGGTTCAGTAGTTGCTCTGTATGTTTAATGgggTTACGCTGGAAGGTC
GATACGCATCACTATGCAATTGTTAGGGCTCCCATATATTTAGGTTCAGTAGTTGCTCTGTATGTTTAATGTTTTTACGCTGtggGGTC

*Lk*ARS-E848 min

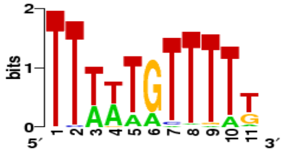

TTCTCCAgggCATAATTTACCAAATGGAATTTTTTTTATGTTTCCTATAATCTACGCGGGTTTTACTATTATTGTTGAAAAAAAAAAGTA
TTCTCCATTTCATAATTTACTgggTGGAATTTTTTTTATGTTTCCTATAATCTACGCGGGTTTTACTATTATTGTTGAAAAAAAAAAGTA
TTCTCCATTTCATAATTTACCAAATGGAATgggTTTTATGTTTCCTATAATCTACGCGGGTTTTACTATTATTGTTGAAAAAAAAAAGTA
TTCTCCATTTCATAATTTACCAAATGGAATTTTTTTTATGgggCCTATAATCTACGCGGGTTTTACTATTATTGTTGAAAAAAAAAAGTA
TTCTCCATTTCATAATTTACCAAATGGAATTTTTTTTATGTTTCCTATAATCgggGCGGGTTTTACTATTATTGTTGAAAAAAAAAAGTA
TTCTCCATTTCATAATTTACCAAATGGAATTTTTTTTATGTTTCCTATAATCTACGCGGGTTTTACgggTATTGTTGAAAAAAAAAAGTA
TTCTCCATTTCATAATTTACCAAATGGAATTTTTTTTATGTTTCCTATAATCTACGCGGGTTTTACTATTATTGTTGAAgggAAAAAGTA

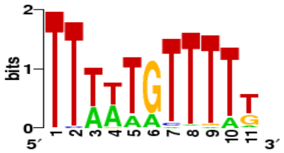

*Lk*ARS-C35 min

GTTCcccCCTGTTTGTTTATTTCAAGTTTGTTATAGCAGATGCAGTTTTAGCGCGGTACAAAAGGCCGAAACTCTGCCAAAAGCATA
GTTCTTTCCTGTTcccTTATTTCAAGTTTGTTATAGCAGATGCAGTTTTAGCGCGGTACAAAAGGCCGAAACTCTGCCAAAAGCATA
GTTCTTTCCTGTTTGTTTATTTCAcccTTGTTATAGCAGATGCAGTTTTAGCGCGGTACAAAAGGCCGAAACTCTGCCAAAAGCATA
GTTCTTTCCTGTTTGTTTATTTCAAGTTTGTTATAGCAcccGCAGTTTTAGCGCGGTACAAAAGGCCGAAACTCTGCCAAAAGCATA
GTTCTTTCCTGTTTGTTTATTTCAAGTTTGTTATAGCAGATGCAGTTcccGCGCGGTACAAAAGGCCGAAACTCTGCCAAAAGCATA
GTTCTTTCCTGTTTGTTTATTTCAAGTTTGTTATAGCAGATGCAGTTTTAGCGCGGTACcccAGGCCGAAACTCTGCCAAAAGCATA
GTTCTTTCCTGTTTGTTTATTTCAAGTTTGTTATAGCAGATGCAGTTTTAGCGCGGTACAAAAGGCCGcccCTCTGCCAAAAGCATA
GTTCTTTCCTGTTTGTTTATTTCAAGTTTGTTATAGCAGATGCAGTTTTAGCGCGGTACAAAAGGCCGAAACTCTGCCAcccGCATA

Figure S3

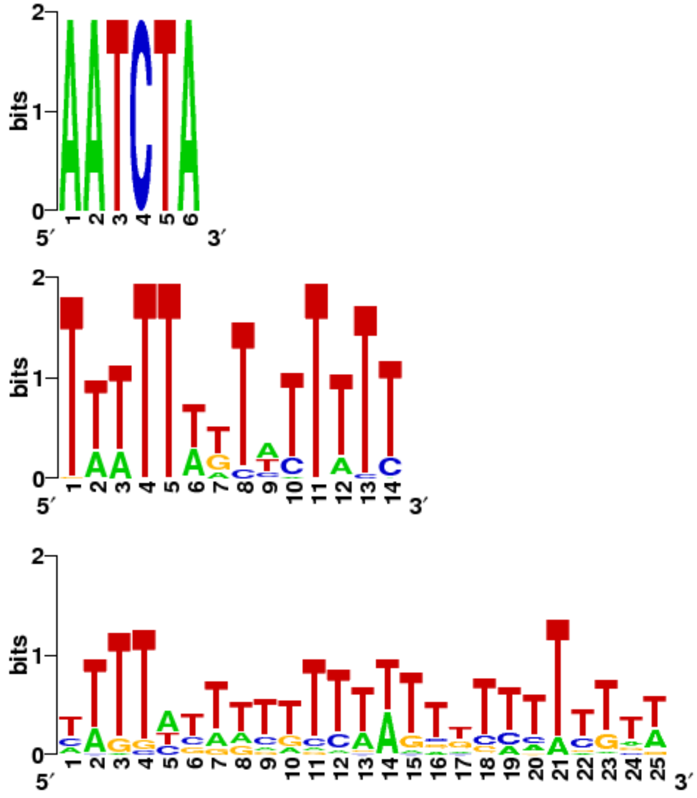

Figure S4

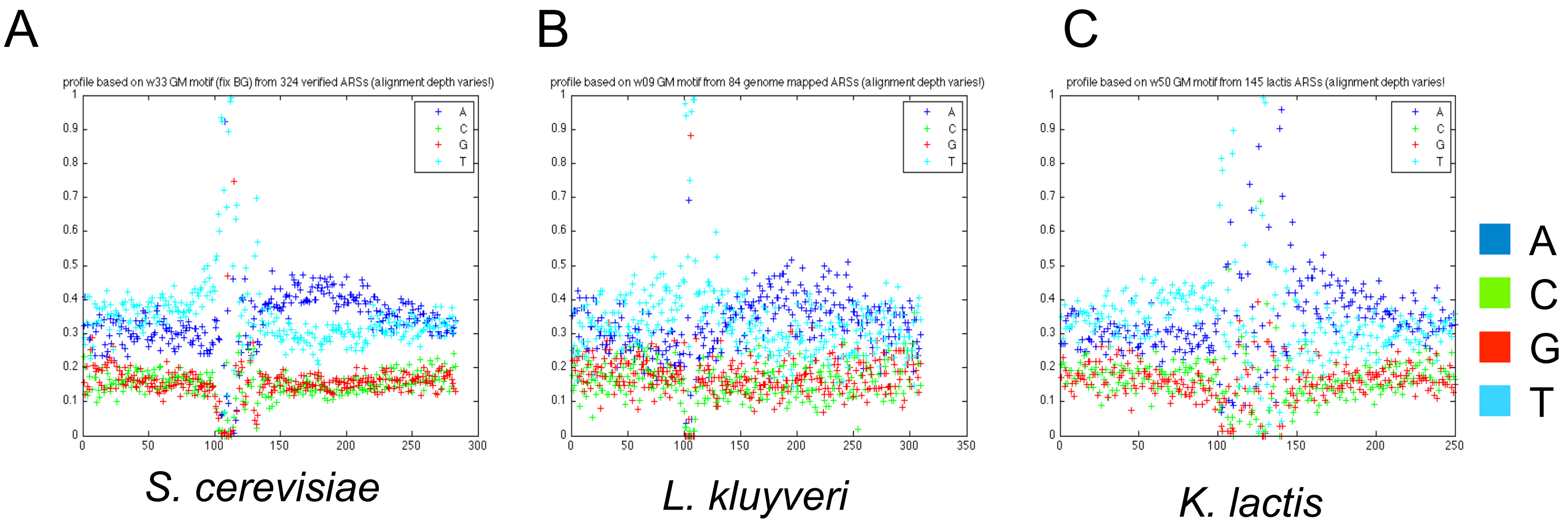
A
profile based on w33 GM motif (fix BG) from 324 verified ARSs (alignment depth varies!)
S. cerevisiae
B
profile based on w09 GM motif from 84 genome mapped ARSs (alignment depth varies!)
L. kluyveri
C
profile based on w50 GM motif from 145 lactis ARSs (alignment depth varies!
K. lactis
A
C
G
T
